# Serum *Aspergillus* Immunoglobulin G as an Independent Biomarker for Extrasinonasal Involvement in Chronic Invasive *Aspergillus* Rhinosinusitis

**DOI:** 10.1093/ofid/ofag059

**Published:** 2026-02-09

**Authors:** Jun-Tian Huang, Ling-Hong Zhou, Wen-Jia Qiu, Hui Tang, Rong-Sheng Zhu, Ying-Kui Jiang, Hua-Zhen Zhao, Zhong-Qing Chen, Li-Ping Zhu

**Affiliations:** Department of Infectious Diseases, Shanghai Key Laboratory of Infectious Diseases and Biosafety Emergency Response, National Medical Center for Infectious Diseases, Huashan Hospital, Fudan University, Shanghai, China; Department of Infectious Diseases, Shanghai Key Laboratory of Infectious Diseases and Biosafety Emergency Response, National Medical Center for Infectious Diseases, Huashan Hospital, Fudan University, Shanghai, China; Department of Infectious Diseases, Shanghai Key Laboratory of Infectious Diseases and Biosafety Emergency Response, National Medical Center for Infectious Diseases, Huashan Hospital, Fudan University, Shanghai, China; Department of Infectious Diseases, Shanghai Key Laboratory of Infectious Diseases and Biosafety Emergency Response, National Medical Center for Infectious Diseases, Huashan Hospital, Fudan University, Shanghai, China; Department of Infectious Diseases, Shanghai Key Laboratory of Infectious Diseases and Biosafety Emergency Response, National Medical Center for Infectious Diseases, Huashan Hospital, Fudan University, Shanghai, China; Department of Infectious Diseases, Shanghai Key Laboratory of Infectious Diseases and Biosafety Emergency Response, National Medical Center for Infectious Diseases, Huashan Hospital, Fudan University, Shanghai, China; Department of Infectious Diseases, Shanghai Key Laboratory of Infectious Diseases and Biosafety Emergency Response, National Medical Center for Infectious Diseases, Huashan Hospital, Fudan University, Shanghai, China; Department of Pathology, Huashan Hospital, Fudan University, Shanghai, China; Department of Infectious Diseases, Shanghai Key Laboratory of Infectious Diseases and Biosafety Emergency Response, National Medical Center for Infectious Diseases, Huashan Hospital, Fudan University, Shanghai, China

**Keywords:** *Aspergillus* IgG antibody, biomarker, chronic invasive aspergillus rhinosinusitis, extrasinonasal involvement, prognostic factors

## Abstract

**Background:**

To investigate the seroprevalence of *Aspergillus* IgG antibodies among patients with chronic invasive *Aspergillus* rhinosinusitis (CIARS) and to assess their prognostic value for extrasinonasal involvement and therapeutic outcomes.

**Methods:**

A total of 132 patients with histopathologically confirmed CIARS were included. Serum *Aspergillus* IgG antibody levels were measured by enzyme-linked immunosorbent assay. Univariate and multivariable analyses were conducted to identify independent predictors of extrasinonasal involvement. To evaluate the prognostic value of antibody monitoring, serial *Aspergillus* IgG levels were assessed, and their association with radiological remission was analyzed.

**Results:**

Among the 132 patients, 50 (37.9%) tested positive for *Aspergillus* IgG antibodies. Seropositivity was significantly higher among patients with extrasinonasal involvement than among those without such involvement (59.2% vs 8.9%; *P* < .001). Multivariable analysis identified positive serum *Aspergillus* IgG antibodies (odds ratio [OR] = 11.28, 95% confidence interval [CI]: 3.67–34.64, *P* < .001), sphenoid sinus involvement (OR = 4.72, 95% CI: 1.89–11.79, *P* < .001), and ethmoid sinus involvement (OR = 5.22, 95% CI: 1.53–17.86, *P* = .008) as independent predictors of extrasinonasal involvement. Serial antibody monitoring was conducted to evaluate treatment outcomes, revealed a significant decrease in *Aspergillus* IgG levels in the radiological and clinical remission groups after antifungal therapy (median = 16.12 NovaTec units [NTU]; interquartile range [IQR] = 12.87–21.96 vs median = 9.21 NTU; IQR = 6.52–13.23; *P* < .001). In contrast, no significant change was observed in the stable disease group.

**Conclusions:**

*Aspergillus* IgG antibody is a promising noninvasive biomarker associated with extrasinonasal invasion and disease progression in CIARS.

## Teaser

*Aspergillus* immunoglobulin G antibodies, a noninvasive serological biomarker, might predict extrasinonasal involvement and monitor therapeutic response in chronic invasive fungal rhinosinusitis, thereby providing valuable prognostic insight and supporting timely antifungal therapy.

## Graphical Abstract

**Graphical Abstract:**
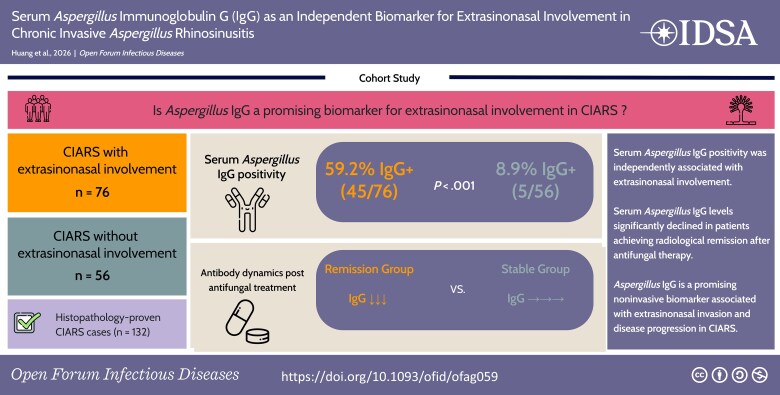
This graphical abstract is also available at Tidbit: https://tidbitapp.io/tidbits/aspergillus-igg-an-independent-biomarker-for-extrasinonasal-and-disease-progression-in-chronic-invasive-aspergillus-rhinosinusitis?utm_campaign=tidbitlinkshare&utm_source=IO

Chronic invasive *Aspergillus* rhinosinusitis (CIARS) is a progressive, life-threatening fungal infection characterized by hyphal invasion of the sinonasal mucosa with extension into adjacent bony or neurovascular structures [1]. CIARS typically presents with nonspecific symptoms, including nasal obstruction, facial pain, and neuro-ophthalmic disturbances. The insidious nature of disease progression, combined with these vague clinical manifestations, often results in diagnostic delay, with a median time to diagnosis exceeding 6 months (range, 4–24 months) [2]. The protracted course of CIARS allows fungal invasion to progress over months to years, ultimately breaching bony barriers and involving extrasinonasal structures [3]. Extrasinonasal involvement is a critical determinant of prognosis in invasive fungal sinusitis (IFS): orbital invasion might precipitate vision-threatening orbital apex syndrome, whereas intracranial dissemination could result in life-threatening cranial neuropathies [4]. Reported mortality rates increase stepwise with anatomical progression, reaching ∼33% with orbital involvement, 50% with skull base invasion, and nearly 100% with cerebral dissemination [5]. Therefore, early diagnosis is critical to prevent irreversible tissue damage and reduce mortality. Current management strategies rely on endoscopic surgical debridement combined with systemic antifungal therapy, typically administered for 6–12 weeks postoperatively [6]. However, therapeutic success largely depends on identifying the disease before irreversible extrasinonasal extension occurs. Consequently, identifying validated predictors of extrasinonasal invasion is essential for effective risk stratification and timely intervention.

CIARS diagnosis currently requires histopathological confirmation of fungal invasion in conjunction with radiographic evidence of bony or soft tissue destruction. Although definitive, these approaches are invasive, labor intensive, and poorly suited for early diagnosis or longitudinal disease monitoring. Serum biomarkers, such as the (1,3)-β-D-glucan (BDG) and galactomannan (GM) assays, are widely used for diagnosing systemic fungal infections; however, their clinical utility in CIARS remains limited. Recent clinical investigations have demonstrated suboptimal diagnostic performance for both assays, with positivity rates of 13.6% for the BDG test and 9.1% for the GM test [7]. These findings underscore the need for more specific, noninvasive biomarkers for CIARS.

*Aspergillus* immunoglobulin G (IgG) antibodies serve as a validated diagnostic biomarker for chronic pulmonary aspergillosis (CPA), demonstrating a specificity of 89.6% (95% confidence interval [CI]: 86.2–92.3) and a positive predictive value of 100% [8, 9]. Given its high specificity in CPA diagnosis, *Aspergillus* IgG antibodies might serve as a reliable biomarker for CIARS, particularly when interpreted in conjunction with clinical and radiological findings. In this cohort study, we analyzed 132 histopathologically confirmed cases of CIARS to assess the utility of serum *Aspergillus* IgG antibodies for risk stratification and prognostic evaluation. Specifically, we aimed to investigate their role in identifying high-risk patients and to evaluate their potential as tools for early detection and monitoring of treatment response.

## METHODS

### Participants and Procedures

This retrospective cohort study was conducted at Huashan Hospital, a national medical center for infectious diseases in Shanghai, China, between January 2017 and June 2025. Adult patients (≥18 years) with histopathologically confirmed CIARS during this period were consecutively included. A confirmed CIARS diagnosis was established based on the following criteria: (1) disease duration exceeding 12 weeks and (2) histopathological evidence of tissue invasion by fungal hyphae consistent with *Aspergillus* species, characterized by dense hyphal aggregates, sparse inflammatory responses, and occasional vascular invasion [10–12]. All pathology specimens were confirmed by an experienced pathologist using standardized diagnostic criteria. Exclusion criteria included acute disease onset (less than 4 weeks) and incomplete clinical data. A total of 132 patients met the inclusion criteria. Patient data were extracted from medical records and included demographic characteristics, predisposing factors, clinical signs and symptoms, laboratory findings, serial radiological assessments, histopathological results, antifungal treatment regimens, and clinical outcomes. Institutional Review Board approval was obtained from the Medical Ethics Committee of Huashan Hospital, Fudan University, Shanghai, China (IRB approval reference number: KY2022-1014). The study was conducted in accordance with the principles of the Declaration of Helsinki.

### Definitions

Orbital involvement in CIARS was defined by the following criteria: (1) radiological evidence of orbital extension and (2) compatible clinical features ophthalmologic symptoms [5]. Orbital apex syndrome was defined as the presence of vision loss due to optic neuropathy and restricted ocular motility, ptosis, or proptosis resulting from involvement of the ocular motor nerves within the orbital apex.

Cerebral involvement in CIARS was defined by radiological evidence of meningeal enhancement or cerebral parenchymal abnormalities consistent with invasive fungal disease (IFD), including cerebral abscesses, parenchymal lesions, ventricular enlargement, or ventriculomegaly [13].

### Serum *Aspergillus* Immunoglobulin G Antibody and Galactomannan Detection

Baseline serum *Aspergillus* IgG antibody levels were measured after histopathologic confirmation and before the initiation of systemic antifungal therapy in all patients. Serum *Aspergillus* IgG antibody levels were measured using a commercial *Aspergillus* IgG enzyme-linked immunosorbent assay kit (IBL International, Hamburg, Germany), according to the manufacturer's predefined thresholds: positive (>11 NovaTec units [NTU]), equivocal (9–11 NTU), and negative (<9 NTU). Each sample was assayed in triplicate. A receiver operating characteristic (ROC) curve analysis was conducted to determine the optimal *Aspergillus* IgG antibody cutoff for predicting extrasinonasal involvement among patients with IgG-positive . Serum GM test was performed using *Aspergillus* GM detection kit (Genobio Pharmaceutical Co. Ltd., Tianjin, China) according to the manufacturer's instructions, with positivity defined as optical density index ≥ 0.5. All serum samples were collected at admission and stored at −80°C until analysis.

### Longitudinal *Aspergillus* Immunoglobulin G Antibody Monitoring and Outcome Assessment

For longitudinal evaluation, follow-up serum *Aspergillus* IgG antibody levels were measured after 3–5 months of continuous antifungal therapy (median: 4 months) in 28 patients without modification of the treatment regimen. Follow-up assessments included physical examination, laboratory testing, and radiological evaluation using paranasal sinus computed tomography and cranial magnetic resonance imaging. Comparative analyses of pretreatment and post-treatment *Aspergillus* IgG levels were performed in relation to clinical and radiological findings. Patients who achieved both symptom resolution and radiological improvement were classified as the remission group, whereas those with persistent or progressive disease were classified as the stable group.

### Statistical Analysis

Continuous variables with a normal distribution are presented as mean ± standard deviation (x ± s), while variables with a non-normal distribution are presented as median interquartile range (IQR). Categorical variables were analyzed using the chi-square test or Fisher's exact test, as appropriate. Continuous variables were analyzed using independent *t*-tests for normally distributed data, with normality assessed using the Shapiro–Wilk test. Univariate analyses were conducted to identify the risk factors associated with extrasinonasal involvement in CIARS. Variables with *P* < .05 in univariate analyses were included in a multivariable binary logistic regression model to identify independent risk factors for extrasinonasal involvement. Results are reported as adjusted odds ratios (ORs) with 95% CIs. A *P* value < .05 was considered statistically significant. ROC curve analyses were performed, and areas under the curve (AUCs) were calculated to assess diagnostic discrimination. The optimal cutoff value was determined using Youden's J statistic (sensitivity + specificity − 1). For longitudinal assessment of *Aspergillus* IgG antibody levels, paired *t*-tests were used to compare pre- and post-treatment IgG levels within each response group after confirming normality using the Shapiro–Wilk test (*P* > .05 for both groups). All statistical analyses were performed using SPSS version 29.0 (SPSS Inc.) or GraphPad Prism version 10.0 (GraphPad Software Inc.).

## RESULTS

### Patient Demographics and Baseline Characteristics

This study included 132 patients with a median age of 56 years (IQR: 49–66). Of these, 56 (42.4%) were male, and 55 (41.7%) had at least 1 predisposing factor. Baseline characteristics, predisposing factors, sinus involvement, and extrasinonasal extension are summarized in Table 1. Among patients with extrasinonasal involvement, orbital involvement was the most common (40.2%, 53/132), with 32 patients (24.2%) presenting with orbital apex syndrome (Table 2). This was followed by cranial involvement (22.0%, 29/132) and mastoid involvement (14.4%, 19/132; Table 3).

**Table 1.** Demographics, Basic Characteristics and Involvements of CIARS Cases

Variable	CIARS Cases (n = 132)	
Male, n (%)	56	(42.4)
Age(y), median (IQR)	56	(49–66)
Predisposing factors, n (%)	55	(41.7)
Diabetes mellitus	35	(26.5)
Glucocorticosteroids**^a^**/immunosuppressants	17	(12.9)
Chronic kidney disease	13	(9.8)
Autoimmune diseases	11	(8.3)
Radiotherapy or chemotherapy	8	(6.1)
Solid malignancy	7	(5.3)
Trauma or destruction of facial structures	5	(3.8)
Hematologic malignancy	4	(3.0)
Liver cirrhosis	2	(1.5)
Paranasal sinuses involvement, n (%)		
Maxillary sinus	109	(82.6)
Ethmoid sinus	108	(81.8)
Sphenoid sinus	71	(53.8)
Frontal sinus	31	(23.5)
Extrasinonasal involvement, n (%)	76	(57.6)
Orbit	53	(40.2)
Cranial	29	(22.0)
Cavernous sinus	26	(19.7)
Mastoid	19	(14.4)
Pterygopalatine fossa	11	(8.3)
Pituitary fossa	8	(6.1)

Abbreviations: CIARS, chronic invasive *Aspergillus* rhinosinusitis; IQR, interquartile range.

^a^Daily prednisone-equivalent doses: median 10 mg/d (IQR, 10–45 mg/d).

**Table 2.** Clinical Manifestation and Sinuses Involvements of CIARS Cases With Orbital Apex Syndrome

Variable	CIARS Cases With Orbital Apex Syndrome (n = 32)	
Manifestations at baseline, n (%)		
Visual impairment	32	(100.0)
Blindness	23	(71.9)
Unilateral	19	(59.4)
Bilateral	4	(12.5)
Impaired eye movement	29	(90.6)
Ptosis	28	(87.5)
Periorbital pain	26	(81.3)
Proptosis	20	(62.5)
Epiphora	3	(9.4)
Paranasal sinuses involvement, n (%)		
Ethmoid sinus	30	(93.8)
Sphenoid sinus	25	(78.1)
Maxillary sinus	24	(75.0)
Frontal sinus	7	(21.9)

Abbreviation: CIARS, chronic invasive *Aspergillus* rhinosinusitis.

**Table 3.** Sinuses Involvement in CIARS Cases with Cranial Involvement

Variable	CIARS Cases With Cranial Involvement (n = 29)	
Paranasal sinuses involvement, n (%)		
Ethmoid sinus	28	(96.6)
Maxillary sinus	24	(82.8)
Sphenoid sinus	22	(75.9)
Frontal sinus	9	(31.0)
Intracranial sites involvement, n (%)		
Temporal lobe	18	(62.1)
Frontal lobe	15	(51.7)
Occipital lobe	2	(6.9)
Hypophysis	2	(6.9)
Cerebral falx	2	(6.9)
Cerebellum	2	(6.9)
Single intracranial site involvement, n (%)	19	(65.5)
Multiple intracranial site involvement, n (%)	10	(34.5)

Abbreviation: CIARS, chronic invasive *Aspergillus* rhinosinusitis.

### Serum *Aspergillus* Immunoglobulin G Antibody and Galactomannan Detection in Chronic Invasive *Aspergillus* Rhinosinusitis

Serum *Aspergillus* IgG antibody levels were measured in all 132 patients, of whom 50 (37.9%) tested positive. Positivity was significantly higher among patients with extrasinonasal involvement than among those with disease confined to the sinonasal region (59.2% [45/76] vs 8.9% [5/56]; *P* < .001). The highest IgG seropositivity rate was observed in patients with orbital apex syndrome (62.5% [20/32]), followed by cranial involvement (62.1% [18/29]) and orbital involvement (60.4% [32/53]) (Supplementary Figure 1A). In contrast, serum GM testing was positive in only 6.8% (9/132) of the patients (Supplementary Figure 1B). To further investigate the potential confounding by immune status, we also performed a stratified analysis in Supplementary Table 1, which summarizes IgG positivity and levels by immune status. However, no significance was identified.

**Figure 1.**
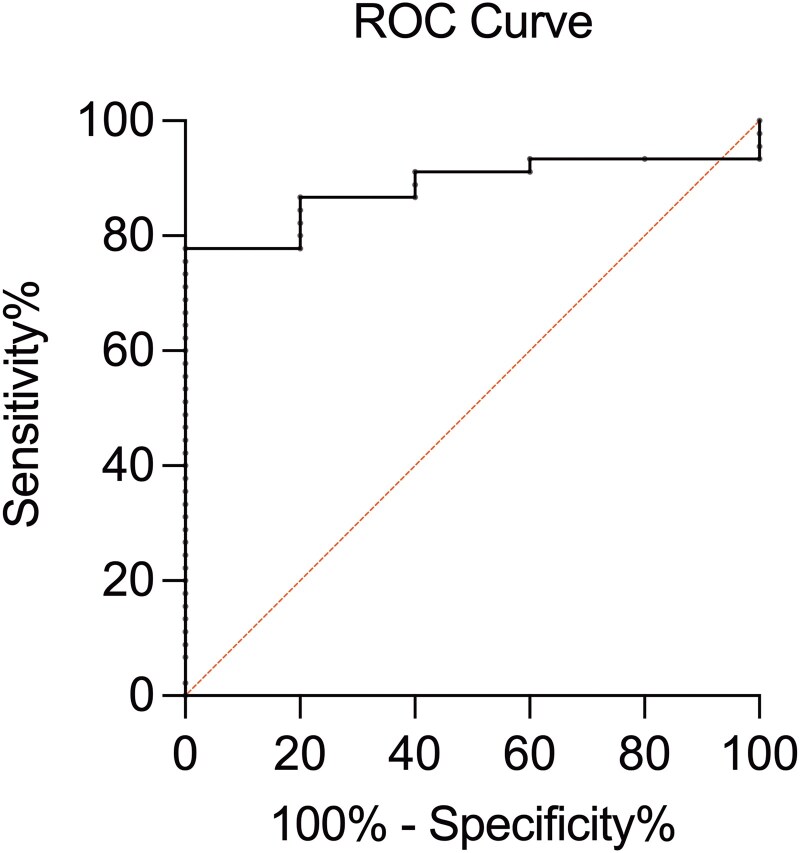
ROC curve distinguishing extrasinonasal involvement from disease confined to the sinonasal region among patients with positive serum *Aspergillus* IgG antibody. The AUC was 0.884 (standard error: 0.049; 95% CI: 0.79–0.98). The optimal cutoff value was 13.08 NTU. Abbreviations: ROC, receiver operating characteristic; IgG, immunoglobulin G; AUC, area under the curve; CI, confidence interval.

ROC curve analysis using Youden's index identified an optimal serum *Aspergillus* IgG cutoff value of 13.08 NTU for distinguishing extrasinonasal involvement from disease confined to the sinonasal region among patients with IgG-positive. This cutoff yielded a sensitivity of 77.8% and a specificity of 100.0% (AUC = 0.884, 95% CI: 0.79–0.98; Figure 1).

### Independent Risk Factors Associated With Extrasinonasal Involvement in Chronic Invasive *Aspergillus* Rhinosinusitis

Univariate analysis revealed several factors associated with extrasinonasal involvement, including male sex (51.3% vs 30.4%; *P* = .017), sphenoid sinus involvement (71.1% vs 30.4%; *P* < .001), ethmoid sinus involvement (90.8% vs 69.6%; *P* = .003), and positive serum *Aspergillus* IgG antibodies (59.2% vs 8.9%; *P* < .001). Positive serum *Aspergillus* IgG antibody (OR 11.28, 95% CI: 3.67–34.64, *P* < .001), sphenoid sinus involvement (OR 4.72, 95% CI: 1.89–11.79, *P* < .001), and ethmoid sinus involvement (OR 5.22, 95% CI: 1.53–17.86, *P* = .008) were identified as independent risk factors for extrasinonasal involvement in CIARS (Table 4).

**Table 4.** Risk Factors Associated With Extrasinonasal Involvement of CIARS Cases

Variable	Univariate Analysis							Multivariable Analysis		
	CIARS Cases with Extrasinonasal Involvement (n = 76)			CIARS Cases without Extrasinonasal Involvement (n = 56)		*P* Value		OR	(95% CI)	*P* Value
Male, n (%)	39	(51.3)		17	(30.4)	.017		2.48	(.96–6.40)	.060
Age(y), median (IQR)	57	(51–68)		56	(48–62)	.246				
Predisposing factors, n (%)	36	(47.4)		19	(33.9)	.123				
Diabetes mellitus	24	(31.6)		11	(19.6)	.128				
Glucocorticosteroids/immunosuppressants	12	(15.8)		5	(8.9)	.251				
Autoimmune diseases	9	(11.8)		2	(3.6)	.109				
Trauma or destruction of facial structures	2	(2.6)		3	(5.4)	.427				
Chronic kidney disease	9	(12.8)		4	(7.1)	.375				
Malignancy	4	(5.3)		3	(5.4)	.981				
Paranasal sinuses involvement, n (%)										
Sphenoid sinus	54	(71.1)		17	(30.4)	<.001		4.72	(1.89–11.79)	<.001
Ethmoid sinus	69	(90.8)		39	(69.6)	.003		5.22	(1.53–17.86)	.008
Maxillary sinus	60	(78.9)		49	(87.5)	.205				
Frontal sinus	22	(28.9)		9	(16.1)	.088				
Time from diagnosis to antifungal treatment > 3 m, n (%)	25	(32.9)		10	(17.9)	.056				
Positive serum *Aspergillus* IgG antibody, n (%)	45	(59.2)		5	(8.9)	<.001		11.28	(3.67–34.64)	<.001

Abbreviations: CIARS, chronic invasive *Aspergillus* rhinosinusitis; OR, odds ratio; CI, confidence interval; IQR, interquartile range; IgG, immunoglobulin G.

### Treatment Outcome

All patients received combined surgical intervention and antifungal therapy. Voriconazole was the most frequently administered agent (n = 124), followed by posaconazole (n = 4), itraconazole (n = 2), isavuconazole (n = 1), and amphotericin B deoxycholate (n = 1). The median duration of antifungal therapy was 6 months (range: 1–47 months). Treatment duration did not differ significantly among antifungal regimens. Notably, delayed initiation of antifungal therapy (> 1 month postoperatively) was confirmed as an independent risk factor for post-surgery recurrence, associated with a significantly higher recurrence rate of 54.8% (23/42) compared with 6.7% (6/90) in patients treated within 1 month (*P* < .001) (Supplementary Table 2).

### Pre- and Post-antifungal Treatment *Aspergillus* Immunoglobulin G Antibody Dynamics and Therapeutic Response

**Figure 2.**
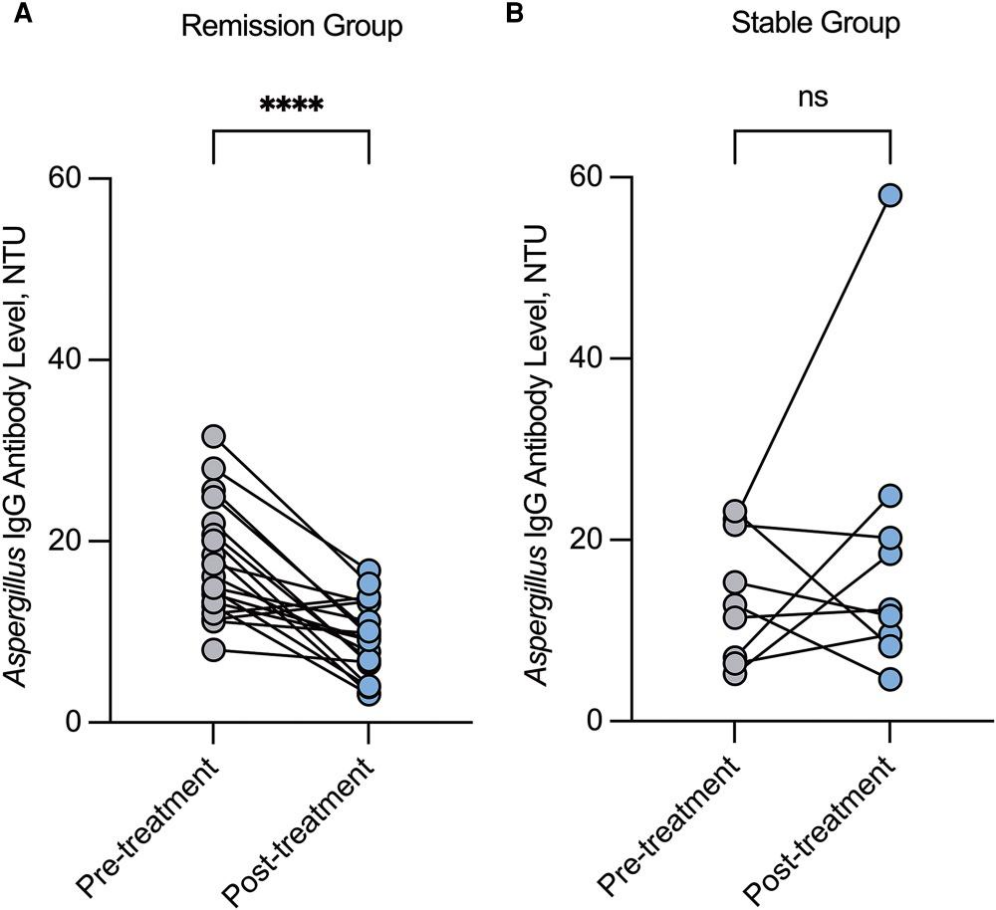
Changes in serum *Aspergillus* IgG levels reflecting therapeutic response in 28 patients with CIARS. *A*, The remission group (n = 19) exhibited a significant decline post-treatment in IgG levels (*P* < .001). *B*, The stable group (n = 9) showed no significant change in IgG levels after treatment (*P* = .345). Abbreviations: CIARS, chronic invasive *Aspergillus* rhinosinusitis; IgG, immunoglobulin G; NS, not significant; NTU, NovaTec units. ****, *P* < .001.

Among the 28 patients with available paired baseline and prespecified follow-up serum *Aspergillus* IgG measurements, 19 (68.9%) achieved combined clinical and radiological remission (remission group). In contrast, 9 (32.1%) demonstrated persistent or progressive disease (stable group). The remission group demonstrated a significant reduction in serum *Aspergillus* IgG antibody levels from baseline, consistent with therapeutic response (median, 16.12 NTU; IQR, 12.87–21.96 vs median, 9.21 NTU; IQR, 6.52–13.23; *P* < .001; Figure 2*A*), corresponding to a median relative decrease of 51.5% (IQR, 24.6%–68.6%) from baseline. In contrast, no significant change in IgG levels was observed in the stable group (median, 12.84 NTU; IQR, 6.71–21.98 vs median, 12.30 NTU; IQR, 8.94–22.56; *P* = .381) (Figure 2*B*).

## DISCUSSION

The findings of this study indicate that patients with CIARS are at increased risk of developing extrasinonasal complications when sphenoid or ethmoid sinus involvement is present. More importantly, *Aspergillus* IgG—which plays a central role in CPA diagnosis [14]—was identified as a novel noninvasive biomarker with potential utility for risk stratification and prognostic assessment in CIARS.

In this study, patients with CIARS and extrasinonasal involvement more frequently exhibited sphenoid or ethmoid sinus involvement, findings that are consistent with prior studies identifying these sinuses as common sources of orbital complications [15–17]. Ethmoid sinus involvement has been reported as an independent risk factor for orbital complications in 43 patients with acute rhinosinusitis (OR = 31.1, 95% CI: 2.3–430.6) [18] Similarly, a retrospective study by Twu et al [19] involving 38 patients demonstrated that IFS originating from the sphenoid sinus (OR = 21.875, 95% CI: 3.295–145.237) or the posterior ethmoid sinus (OR = 14.0, 95% CI: 1.234–158.844) was more likely to disseminate to the orbital region . Although sphenoid and ethmoid sinus involvement have previously been identified as risk factors for orbital complications, this study is the first to demonstrate their association with broader extrasinonasal involvement, including cranial, orbital, and mastoid extension. These findings indicate that clinicians should remain vigilant for the development of multiple extrasinonasal complications in patients with CIARS who present with sphenoid or ethmoid sinus involvement.

Diagnostic challenges in CIARS remain substantial. The disease is frequently associated with orbital apex syndrome, which could lead to visual impairment or blindness, and intracranial extension might be fatal. However, establishing a definitive diagnosis in routine clinical practice is often difficult, as it typically relies on histopathological confirmation of fungal hyphae invading mucosal or submucosal tissues, vasculature, or bone. Galactomannan testing, one of the most commonly used assays for fungal infections, has been recommended as a primary screening tool for the early diagnosis of IFD. However, in non-neutropenic patients, GM testing demonstrates limited diagnostic performance, with a sensitivity of 23.1% and a specificity of 76.1% in individuals with nonhematologic disorders [20]. Moreover, GM testing was negative in 25 patients with histopathologically confirmed fungal ball of the paranasal sinuses [7], a finding consistent with our results.

Therefore, there is an urgent need to identify methods that enable the early and rapid detection of extrasinonasal involvement. Given the indolent course of CIARS, we evaluated the diagnostic performance of *Aspergillus*-specific IgG antibody testing in this patient population. *Aspergillus*-specific IgG antibodies are a well-established marker of infection in CPA and are recommended for clinical use in international guidelines [8]. Detection of *Aspergillus*-specific IgG antibodies is simple, inexpensive, and readily applicable in most clinical settings [21]. However, prior to this study, IgG testing had been used primarily for CPA, allergic bronchopulmonary aspergillosis and allergic fungal rhinosinusitis [22–24]. To date, only Hung et al reported that *Aspergillus*-specific IgG antibodies could be used in the diagnosis of chronic rhinosinusitis (CRS) [24], though their study was limited to type 2 CRS and extrasinonasal involvement was not evaluated. Notably, our study demonstrated that IgG seropositivity was substantially more prevalent among patients with CIARS and extrasinonasal involvement, suggesting that IgG detection could be a promising biomarker for prognostic assessment in CIARS. Thus, clinicians should remain alert to the possibility of extrasinonasal involvement when patients with CIARS present with relevant clinical symptoms and a positive *Aspergillus* IgG result.

Although not formally recommended by guidelines, *Aspergillus* IgG quantification has been used to diagnose CPA in large cohorts [25, 26]. Reported optimal diagnostic cutoffs vary by assay: 1.5 AU/mL for Bio-Rad (93% sensitivity, 98% specificity), 25 mg/L for Immulite (93% sensitivity, 99% specificity), 50 mg/L for ImmunoCAP (84% sensitivity, 96% specificity), and 50 U/mL for Serion (84% sensitivity, 91% specificity) [25]. However, positive results have been reported in approximately one-third of healthy individuals, underscoring the need to interpret *Aspergillus* IgG levels in conjunction with radiological findings [26]. In this study, we included patients with CIARS diagnosed based on histopathological and radiological findings. We further demonstrated that an *Aspergillus* IgG level > 13.08 NTU might serve as a promising indicator of extrasinonasal involvement at diagnosis, offering a potential strategy for risk stratification and treatment monitoring.

The mainstay of treatment for CIARS is surgical debridement combined with timely systemic antifungal therapy. Although some authors have suggested that surgery alone might be sufficient [27], our findings indicate that systemic antifungal treatment is an essential component of CIARS management. Since histopathological confirmation does not exclude an invasive process [19], delays in antifungal initiation are associated with an increased risk of CIARS progression. Therefore, our study indicates that patients with fungal rhinosinusitis and clinically evident neuro-orbital complications might still benefit from postoperative systemic antifungal therapy.

Evidence from multiple CPA cohorts support the potential value of monitoring *Aspergillus* IgG antibody levels to assess treatment response. Clinical management statements for CPA also acknowledge that serologic markers might serve as a valuable adjunct to clinical and radiological assessments during follow-up [28]. Li et al [9] examined longitudinal changes in *Aspergillus* IgG levels during antifungal therapy in CPA. They reported that serum IgG levels decreased in patients who responded to treatment but increased in those with subsequent disease progression, indicating that temporal IgG trends might reflect long-term therapeutic effectiveness. Similarly, Hung et al found an association between *Aspergillus*-specific IgG antibodies and symptom scores evaluated by the Sino-Nasal Outcome Test-22 [24]. Consistent with these observations, our study demonstrated that serial *Aspergillus* IgG monitoring in responders (66.7% of the follow-up cohort) was associated with significant declines in IgG levels, suggesting that IgG dynamics might serve as a potential marker of treatment response in CIARS.

This retrospective, single-institution study might represent the first cohort to examine the factors associated with extrasinonasal involvement in CIARS. Nevertheless, several limitations should be acknowledged. Given the rarity of CIARS, the subgroup sample sizes were relatively small. The retrospective design and incomplete data also limit the robustness of the findings. Additionally, cohort inclusion required histopathological confirmation, which might have preferentially selected patients undergoing surgery and potentially underrepresented milder disease presentations. Prospective, multicenter studies are required to validate the generalizability of these findings, including the transportability of the ROC-derived cutoff across assays and clinical settings. Future investigations should standardize sampling time points, incorporate longer-term serial measurements, and apply harmonized outcome definitions with prespecified analyses stratified by immune status. Systematic microbiological characterization, including species-level identification, would further strengthen the interpretation of the results and their clinical applicability.

## CONCLUSION

This study demonstrates that serum *Aspergillus* IgG is an independent predictor of extrasinonasal involvement in CIARS. Serial IgG measurements could help evaluate treatment responses and support their use in long-term disease surveillance. Incorporating *Aspergillus* IgG testing into clinical practice might enable earlier risk recognition and facilitate timely intervention.

## Supplementary Material

ofag059_Supplementary_Data

## Supplementary Data

Supplementary materials are available at *Open Forum Infectious Diseases* online. Consisting of data provided by the authors to benefit the reader, the posted materials are not copyedited and are the sole responsibility of the authors, so questions or comments should be addressed to the corresponding author.
