# Supplementary material for: Serum Aspergillus Immunoglobulin G as an Independent Biomarker for Extrasinonasal Involvement in Chronic Invasive Aspergillus Rhinosinusitis
Source: Open Forum Infect Dis. 2026 Feb 9;13(2):ofag059. doi: 10.1093/ofid/ofag059 (PMC12951068; doi:10.1093/ofid/ofag059)
Supplement: ofag059_Supplementary_Data [file ofag059_supplementary_data.docx]

**SUPPLEMENTARY APPENDIX**

**Table of Contents**

| **Supplementary Figure 1.** Serum *Aspergillus* IgG antibody detection and galactomannan test results in different CIARS involvement groups. | Page 2 |
| --- | --- |
| **Supplementary Table S1.** Serum *Aspergillus* IgG antibody positivity rate stratified by immune status of CIARS cases | Page 3 |
| **Supplementary Table S2.** Risk factors associated with post-surgery recurrence of CIARS cases | Page 4 |

**SUPPLEMENTARY FIGURE**


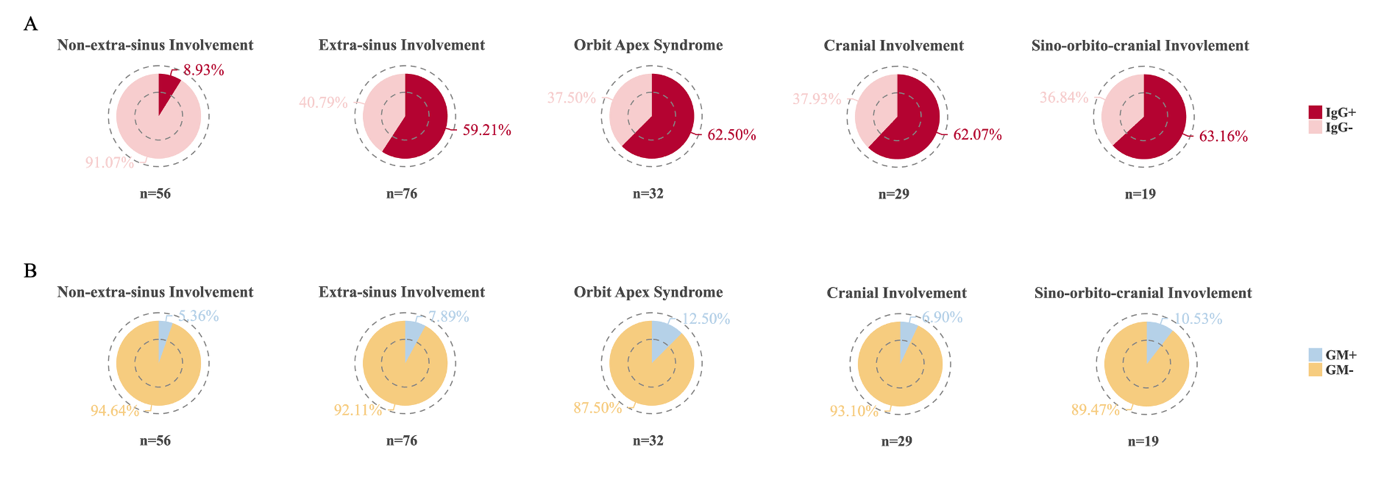


**Supplementary Figure S1.** Serum *Aspergillus* IgG antibody and GM positivity rates in patients with different CIARS involvement. (A) *Aspergillus* IgG antibody results in different CIARS involvement groups. (B) GM tests results in different CIARS involvement groups. Abbreviations: GM, galactomannan; CIARS, chronic invasive *Aspergillus* rhinosinusitis.

**SUPPLEMENTARY TABLE**

**Supplementary Table S1. Serum *Aspergillus* IgG Antibody Positivity Rate Stratified by Immune Status of CIARS Cases**

| Variable | Immunocompromised^a^ | | Non-immunocompromised | | *P* Value |
| --- | --- | --- | --- | --- | --- |
|  |  | (n = 13) |  | (n = 119) |  |
| Extrasinonasal involvement, n (%) | 10 | (65.5) | 66 | (51.9) | .236 |
| Positive serum *Aspergillus* IgG antibody, n (%) | 5 | (41.8) | 45 | (35.1) | >.999 |
| Serum *Aspergillus* IgG antibody level (NTU), median (IQR) | 8.94 | (5.39-13.33) | 7.99 | (5.28-14.14) | .363 |

^a^Immunocompromised patients were identified according to the EORTC/MSGERC 2020 consensus. Including hematologic malignancy(n = 4), prolonged systemic corticosteroid exposure (therapeutic dose of ≥0.3 mg/kg corticosteroids for ≥3 weeks in the past 60 days, n = 4), recent treatment with calcineurin inhibitors (n = 4) and lymphocyte-specific monoclonal antibodies (n = 1).

Abbreviations: CIARS, chronic invasive *Aspergillus* rhinosinusitis; NTU, NovaTec units; IQR, interquartile range.

**Supplementary Table S2. Risk Factors Associated with Post-surgery Recurrence of CIARS Cases**

| Variable | Univariate Analysis | | | | | | | |  | | Multivariable Analysis | | | | | |
| --- | --- | --- | --- | --- | --- | --- | --- | --- | --- | --- | --- | --- | --- | --- | --- | --- |
|  | CIARS Cases with Post-surgery Recurrence^a^ | |  | CIARS Cases without Post-surgery Recurrence | | | *P* Value | |  | | OR | | (95% CI) | | *P* Value | |
|  |  | (n = 29) |  |  | (n = 103) |  | |  | |  | |  | |  | |  |
| Male, n (%) | 15 | (51.7) |  | 41 | (39.8) | .291 | |  | |  | |  | |  | |  |
| Age(y), median (IQR) | 54 | (42-63) |  | 56 | (50-67) | .094 | |  | |  | |  | |  | |  |
| Predisposing factors, n (%) | 9 | (31.0) |  | 46 | (44.7) | .208 | |  | |  | |  | |  | |  |
| Paranasal sinuses involvement, n (%) |  |  |  |  |  |  | |  | |  | |  | |  | |  |
| Sphenoid sinus | 26 | (89.7) |  | 83 | (80.6) | .405 | |  | |  | |  | |  | |  |
| Ethmoid sinus | 25 | (86.2) |  | 83 | (80.6) | .594 | |  | |  | |  | |  | |  |
| Maxillary sinus | 17 | (58.6) |  | 54 | (52.4) | .674 | |  | |  | |  | |  | |  |
| Frontal sinus | 8 | (27.6) |  | 23 | (22.3) | .621 | |  | |  | |  | |  | |  |
| Extrasinonasal involvement, n (%) | 22 | (75.9) |  | 54 | (52.4) | .033 | |  | | 2.65 | | (0.90-7.782) | | .077 | |  |
| Delayed antifungal initiation (> 1-month post-surgery), n (%) | 23 | (79.3) |  | 19 | (18.4) | < .001 | |  | | 16.49 | | (5.81-46.82) | | < .001 | |  |

^a^Defined as the recurrence of fungal rhinosinusitis lesions before the initiation of postoperative antifungal therapy.

Abbreviations: CIARS, chronic invasive *Aspergillus* rhinosinusitis; OR, odds ratio; CI: confidence interval; IQR, interquartile range.
